# Simulation Analysis of Porthole Die Extrusion Process and Die Structure Modifications for an Aluminum Profile with High Length–Width Ratio and Small Cavity

**DOI:** 10.3390/ma11091517

**Published:** 2018-08-23

**Authors:** Zhiwen Liu, Luoxing Li, Shikang Li, Jie Yi, Guan Wang

**Affiliations:** 1 School of Mechanical Engineering, University of South China, Hengyang 421001, China; 2 State Key Laboratory of Advanced Design and Manufacture for Vehicle Body, Hunan University, Changsha 410082, China; kangkangli2010@163.com (S.L.); 15111270111@163.com (J.Y.); belonging1024@126.com (G.W.)

**Keywords:** aluminum profile, porthole die, small mandrel, ALE formulation, die structure modifications, simulation

## Abstract

The design of a porthole die is one of the key technologies for producing aluminum profiles. For an aluminum profile with high length–width ratio and small cavity, it is difficult to control the metal flow through porthole die with the same velocity to ensure the die’s strength. In the present study, the porthole die extrusion process of aluminum profile with small cavity was simulated using HyperXtrude 13.0 software based on ALE formulation. The simulation results show for the traditional design scheme, the metal flow velocity in porthole die at every stage was severely not uniform. The standard deviation of the velocity (SDV) at the die exit was 19.63 mm/s. The maximum displacement in the small mandrel was 0.0925 mm. Then, aiming at achieving a uniform flow velocity and enough die strength, three kinds of die structure modifications for the porthole die were proposed. After optimization, desired optimization results with SDV of 0.448 mm/s at the die exit and small mandrel deflection were obtained. Moreover, the temperature uniformity on the cross-section of die exit, welding pressure, and die strength were improved greatly. Finally, the optimal porthole die was verified by the real extrusion experiment. A design method for porthole die for aluminum with a high length–width ratio and small cavity was proposed, including sunken port bridges to rearrange the welding chamber in upper die, increasing the entrance angle of portholes, introducing the baffle plate, and adjusting the bearing length.

## 1. Introduction

Aluminum alloys are ideal lightweight material for automotive, rail transportation, communication, and aerospace applications, due to their low density, high specific strength, good corrosion resistance, and good recycling ability [1,2]. With the rapid development of extrusion technology, the demand for aluminum profiles becomes more and more important due to the use of space-frame constructions in auto body and high-speed train. The design of extrusion die is one of the key technologies for producing aluminum profiles. A well-designed extrusion die should have favorable metal flow behavior in porthole die to ensure the extruded profiles with uniform flow velocity [3,4]. Otherwise, the extruded profiles can easily be subjected to distortion, thinning, or bending along the extrusion direction. In addition, if the porthole die is not well designed, the mandrel, port bridges, and die bearing maybe generate elastic or plastic deformation under non-uniform pressure and metal velocity distribution, which leads to die scrap and a large size error of products. For the aluminum profiles with high length–width ratio and small cavity, it is difficult to control the metal flow through porthole die with uniform velocity owing to the complicated affecting factors, such as the die structures and process parameters.

Due to the real extrusion production being carried out in the closed container and porthole die, it is practically impossible to measure the temperature, exit velocity distribution, and welding pressure which determine the profile quality. The conventional die design for hollow profiles is generally based on the expertise and practice experience of die designers or expensive plant trials. Thus, it is not easy to guarantee the quality and performance of extruded profiles. To avoid or reduce costly extrusion trials, accurate finite element (FE) simulation can be carried out to investigate the metal flow, extrusion load, and temperature distribution during the whole extrusion cycle, and many other process-related issues [5,6]. By means of FE simulations, the extrusion defects could be forecasted in advance, thereby allowing immediate modification on the die structures and process parameters before production. Kim et al. [7] investigated the non-steady state extrusion process of Al alloy tubes through 3D-FE simulation and analyzed the influence of process parameters on welding pressure, metal flow, and surface defects. Chen et al. [3] proposed a logical and effective route for designing the multi-hole porthole die. The major design steps include finding the optimal position of die orifices, reasonable design of the welding chamber levels, and adjusting the die bearings. Lee et al. [8] investigated the influence of welding chamber shape on die deformation and material flow during condenser tubes extrusion by means of numerical simulation. With the decrease of welding chamber height, the bearing deflection, and dead zone increase. Donati et al. [9] examined the effect of feeder dimension on the weld strength and distortion of extruded profiles under different processing conditions. Bigger feeder dimension allows higher extrusion speed and wider range of process parameters. Liu et al. [10] have optimized the porthole die of hollow thin-walled aluminum profiles using arbitrary Lagrangian–Eulerian formulation (ALE) algorithm. An optimization design was proposed to obtain a uniform metal flow velocity at bearing exit. Zhang et al. [11] investigated the effects of extrusion speed on metal flow, weld strength, extrusion load, etc. for a hollow aluminum profile. It was found that an optimal extrusion speed exists for exit velocity distribution. As extrusion speed increases, seam weld quality and extrusion load increase. Sun et al. [12] proposed an optimization method for the second-step chamber for a condenser tube extrusion die based on the response surface method and genetic algorithm. The optimal shape and height of the second-step chamber were obtained. Zhao et al. [13] optimized the structure parameters of baffle plates near the die orifice for aluminum profiles extrusion to balance the metal flow. Through multiple adjustments, a uniform flow velocity at die exit is obtained. Xue et al. [14] investigated the influence of key die structures such as the billet buckle angle, the porthole bevel angle, the depth of the welding chamber, and the type of bridge on the metal flow balance during multi-output porthole extrusion process through the use of thermo-mechanical modeling combined with the Taguchi method. Gagliardi et al. [15] investigated the influences of geometric variables of porthole die on the extrusion load, the maximum pressure inside the welding chamber, and the material flow homogeneity by ANOVA technique. Then, the Grey relational analysis was introduced to determine the optimal combination of geometric parameters according to specific process needs. Chen et al. [16] designed a porthole die of hollow aluminum profile based on the theory of metal plastic forming and investigated the effect of height and shape of baffle plate on the metal flow velocity in porthole die. Koopman et al. [17] presented an accurate 2D method to simulate the filling of porthole die at the startup of extrusion process based on the pseudo concentration technique. The effect of the location and velocity of flow front on the die deformation was investigated. Lee et al. [18] developed a new porthole extrusion die for improving the welding pressure in the welding chamber by using numerical analysis.

Based on the above literature data, many studies have been done to investigate the metal flow behavior in porthole dies and optimize the die structures and process parameters. However, the research objects were mainly focused on the hollow profiles with simple cross-sections or large cavities. Little research has been reported on the extrusion process or die design for aluminum profiles with high length–width ratio and small cavity; as a result, there are few related documents to be referred for practical production. Therefore, in the present study, the steady-state porthole die extrusion process of a typical hollow profile with the length–width ratio of 42.6 and a small cavity of *Φ* 4.04 mm was simulated using HyperXtrude software based on ALE formulation. Firstly, the metal flow behavior in the porthole die and die strength for the traditional design scheme were investigated. Then, aiming at achieving a uniform flow velocity and enough die strength, three kinds of die structure modifications for the porthole die were proposed. After modifications, desired simulation results with uniform metal flow velocity at die exit and small die deformation were obtained. By such systematic study, a design route of porthole die for the aluminum profile with high length–width ratio and small cavity was proposed to the die designers.

## 2. Porthole Die Design and Simulation Procedures

### 2.1. Traditional Design Scheme and Geometry Modeling

**Figure 1:**
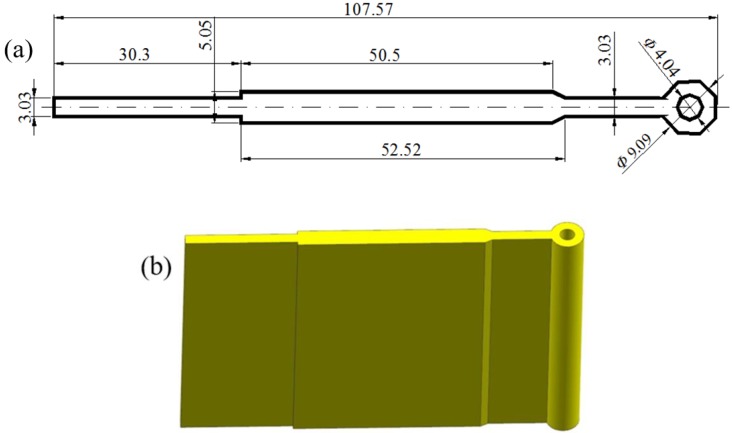
Geometric model of aluminum profile with small cavity: (**a**) 3D shape and (**b**) cross-section. (unit: mm).

Figure 1 shows the shape and size of cross-section for the aluminum profile with high length–width ratio and small cavity studied in this work. It had a length of 107.57 mm in the horizontal direction and a minimum wall thickness of 2.525 mm. The cross-sectional area was 454.75 mm^2^, with the ratio of length to width up to 42.6. Thus, the metal flow behavior in porthole die is quite complex and it is a challenging task to obtain a uniform velocity distribution on the cross-section of die exit. In addition, the profile had a small cavity with diameter of 4.04 mm at the outer end of profile, which further aggravates the difficulty of extrusion.

**Figure 2:**
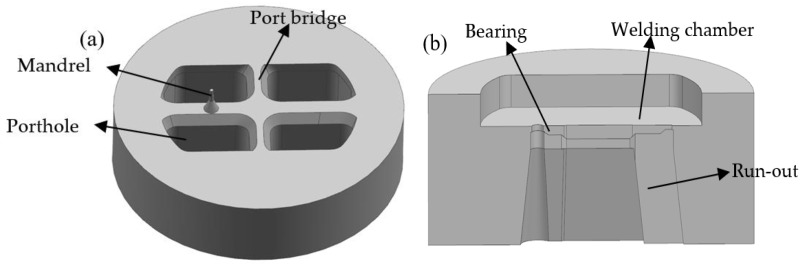
3D models of the traditional designed die with the welding chamber designed in lower die: (**a**) the upper die and (**b**) the lower die.

Figure 2 shows the simplified 3D geometry models of porthole die for the traditional design scheme. Four potholes were arranged symmetrically on the die surface to assign material reasonably. The outer diameter of the porthole die was 250 mm and the heights of upper and lower die were 102 and 64 mm, respectively. A mandrel was utilized to form the internal contour of small cavity and was supported by the interlaced form of port bridges. The widths of port bridges in the transverse and longitudinal directions were 26 and 18 mm, respectively. A chamfering 40° was designed in the bottom of port bridges to decrease the metal flow resistance. The lower die was comprised of the welding chamber, die orifice, die bearing, and run-out, as shown in Figure 2b. The welding chamber with a height of 18 mm was utilized to reweld the metal streams splitting by port bridges under the effect of high pressure and temperature. The die bearing was used to form the outer contour and size of profile and adjust the exit flow velocity of metal. The run-out near the die exit was designed to avoid scratching the extruded profile. Besides, the die orifice was amplified 1.01 times the theoretical size to compensate for shrinkage during cooling.

### 2.2. Establishment of 3D-FE Model

**Figure 3:**
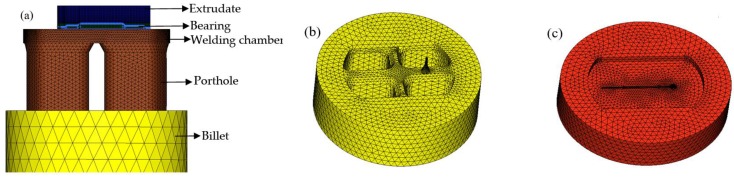
Mesh generation of (**a**) the flow domains, (**b**) upper die, and (**c**) lower die in FE model.

There are three methods can be adopted to simulate the porthole die extrusion by FE methods. The transient updated Lagrangian (UL) formulation, in which the FE meshes are attached to the deforming workpiece, is able to capture the material flow in a very intuitive way [19]. However, the meshes are prone to distortion in large deformation regions and the local remeshing is inevitable, which makes it incompatible with the demand of the efficient and accuracy. Besides for UL formulation, the weld problem between adjacent metal streams is difficult to solve. The steady state Eulerian (SS) formulation [20], where the FE meshes are fixed in space, is fast but the thermal mechanical stationarity may not be well established actually and cannot provide any transient information. The key idea of ALE formulation is to separate the material and mesh displacements [21], thus the mesh distortion and frequent remeshing in UL formulation is avoided. Also, the shortcoming of SS formulation can also be eliminated since the procedure is essentially incremental. In recent years, the application of ALE formulation in the simulation of porthole die extrusion process has been used even more extensively [10,11,12,13,14]. Therefore, in this study, the 3D-FE model for simulating the porthole die extrusion process of aluminum profile with small cavity was established using HyperXtrude software-based ALE formulation. The computational domains of the metal through the container and porthole die was built first by CATIA V5R20 software, then input into the HyperXtrude software in STEP format. Before meshing the flow domains, it is necessary to manually clean up the tiny entities of geometric model that influence seriously the size and quality of mesh generating. Figure 3 shows the 3D-FE models for the flow domains, and upper and lower dies with initial elements. For convenience of meshing and establishing the boundary conditions, the metal through extrusion tools was divided into five different components. The pentahedron mesh type was used in the components of extruded profile and die bearing, while the tetrahedron mesh type was adopted in other components. To improve the simulation accuracy and save computer source, the five components were allocated for different mesh sizes according to the amount of local deformation. The mesh size in the component near the die orifice which suffers a severe shear deformation was set to be 0.5 mm to have at least four nodes in the profile thickness, while the mesh size in the welding chamber, portholes, and billet were about 2, 5, and 20 mm, respectively. Moreover, in order to analyze the die strength, the porthole die also need to be meshed, and tetrahedron element type was used. The mesh sizes of the upper and lower dies were about 0.5–10 mm. The deformation analysis of the dies was carried out by using HyperXtrude solver’s coupled flow, thermal, and stress analysis. The location at the outer diameter of porthole die and die exposed region were specified with X, Y, Z displacement = 0, while in other region of porthole die X, Y, Z traction = 0 is specified to allow the die to freely move. The total number of elements was about 800,000. After modeling, the FE simulations were performed in the DELL T7610 workstation.

In FE simulations, aluminum alloy AA6063 and H13 steel were utilized to material for the billet and porthole die, respectively. The physical properties of AA6063 and H13 are listed in Table 1. The H13 steel has high hardness, thermal stability, and good abrasion resistance, which can withstand to be repeatedly heated and cooled. To simulate the metal flow behavior and analyze the die deflection, the billet was defined as thermoplastic material and the porthole die was defined as elastic material. The Sellars–Tegart model was implemented in FE simulations to describe the flow stress behavior of AA6063 aluminum alloy. For this model, the flow stress $\bar{\sigma }$ is given by

(1) σ¯=1βsinh,−1(ZA)1n,

where *β*, *A*, *n* are the material parameters, which is independent of temperature and can be determined by fitting to the flow stress dates, *Z* is the Zener–Hollomon parameter, expressed by

**Figure 4:**
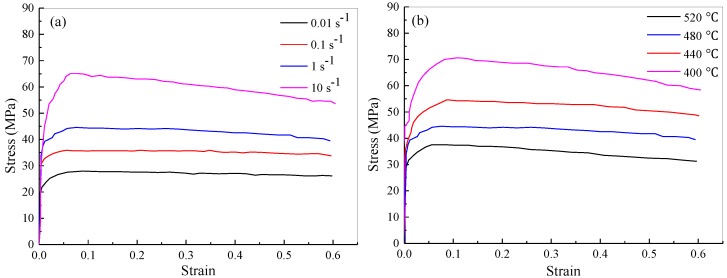
True stress–strain curves of AA6063 aluminum alloy: (**a**) at different strain rates in 480 °C; (**b**) at different temperatures in strain rate 1 s^–1^.

(2) Z=ε˙¯eQRT,

where *R* is the universal gas constant, *T* is the absolute temperature, $\bar{\dot{\varepsilon }}$ is the effective strain rate, and *Q* is the activation energy for deformation. Hot compression experiments were performed using a Gleeble-1500 thermomechanical simulator to obtain stress–strain curves over a temperature range from 400 to 520 °C and strain rate range from 0.01 to 10 s^–1^ with a true strain of 1 (Figure 4). Those flow stress dates obtained were then corrected for flow softening due to deformation heating during hot compression tests, and then were used to determine the material parameters. For AA6063 aluminum alloy used in the present simulations, the parameters in Equations (1) and (2) were as follows: *Q* = 1.71 × 10^5^ J/mol, *A* = 1.18 × 10^9^ s^–1^, *β* = 3.66 × 10^–8^ m^2^/N, *n* = 7.89, *R* = 8314 J/(mol·k).

The process parameters and thermal boundary conditions used in simulations were identical to those in actual extrusion production, as shown in Table 2. The diameter of container was 210 mm and the extrusion ratio (area of container/area of profile) was 75.8, which belongs to the range of large extrusion ratio. The temperature of billet was 480 °C and the temperature of porthole die was 50 °C lower than that of billet. The speed of extrusion ram was 2 mm/s. To obtain accurate simulation results, reasonable friction and heat transfer conditions at the billet/tools interfaces should be adopted. The heat transfer coefficient between the billet and porthole die was set to be 3000 W/m^2^·°C [22,23]. When the extrusion temperature was greater than 400 °C, the friction type at the interface between the billet and porthole dies (except die bearing) was considered to be sticking friction [24]. However, at the interface between the billet and die bearing was defined as sliding friction and the friction coefficient was set to be 0.3 [7].

## 3. FE Simulation of Traditional Die Design

The principal design goal of porthole die is to obtain a uniform exit velocity and high die strength. At the condition of non-uniform metal flow, the billet is extruded out from the die orifice with different velocities throughout its cross-section, resulting in the defects of profile such as bending, twisting, thickness reduction, etc. Thus, the exit velocity uniformity should be highly valued by the die designers. To evaluate quantitatively the velocity distribution of profile at die exit, the standard deviation of velocity (SDV) was introduced, which is defined by

(3) $$SDV=\sqrt{\frac{\sum_{i=1}{\left(v_{i}-\bar{v}\right)}^{2}}{n}},$$

where *v_i_* is the normal velocity for node *i*, $\bar{v}$ is the average velocity, and *n* is the nodes number. In order to obtain a more actual velocity distribution, the velocity values of all nodes on the cross-section of extruded profile were extracted. The smaller the value of SDV, the better the extruded quality is.

**Figure 5:**
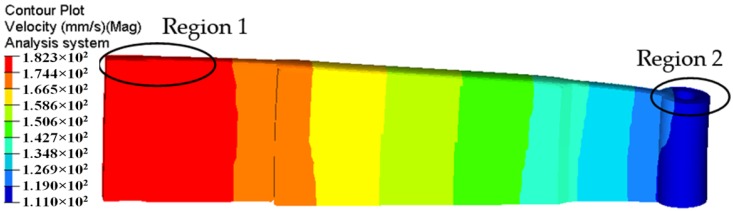
Velocity distribution in the extruded profile for the traditional die design.

The deformation and exit velocity distribution of extruded profile for the traditional die design is shown in Figure 5. It is clearly that the metal flow velocity on the cross section of die exit is extremely nonuniform. From the solid end of the profile (See region 1) to the small cavity (See region 2), the flow velocity of material gradually decreases. The maximum velocity in Region 1 is 182.3 mm/s and minimum velocity in Region 2 is only 111.0 mm/s, which deviates seriously from the theoretical value of 151.8 mm/s. The calculated value of SDV reaches 19.63 mm/s, which indicates a very poor velocity distribution. Owing to the large nonuniformity of exit velocity distribution, the region with high velocity continuously squeezes that with low velocity, consequently causing the extruded profile distorted or bent along its length.

**Figure 6:**
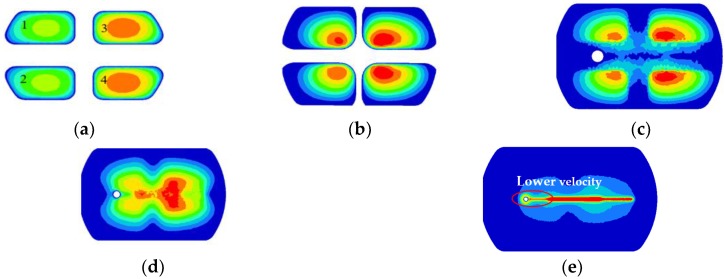
Metal flow at different stages during the whole extrusion process: (**a**) velocity distribution at the entrance of the portholes; (**b**) velocity distribution in the portholes, (**c**) metal flowing the welding chamber, (**d**) metal flowing in the mid-height welding chamber, and (**e**) velocity distribution at the entrance of the die orifice.

Figure 6 shows the velocity distributions of four sections extracted from the entrance of portholes to the bottom of welding chamber along the extrusion direction, which reflects the material flow status in porthole die during the whole extrusion process. Figure 6a shows the metal flow state at the inlet of four portholes. The contours drawn in the figures show the velocities scalar values on the specified surfaces perpendicular to the extrusion direction. Blue and red represent the minimum and maximum velocity values, respectively. The same as below. It is clear from Figure 6a that the metal flow faster in Portholes 3 and 4 than in Portholes 1 and 2, which causes a large velocity difference in the Regions 1 and 2 (See Figure 5). In addition, the metal in the center of every porthole moves faster than that at the periphery. This is mainly because of an intense sticking friction on the portholes wall. Figure 6b shows the material flow state in four portholes. By comparison with Figure 6a, a reducing velocity gradient in each porthole is observed, which is also due to the increasing friction resistance between the billet and portholes. The intense friction on the porthole wall prevents the billet skin from entering the extruded profiles. Figure 6c shows the material flow state at the entrance of welding chamber. There are four dead metal zones under the port bridges where the velocities are nearly zero. Figure 6d shows the velocity distribution in the mid-height of welding chamber. The welding chamber is filled by the barreling legs, and the two adjacent metal streams rejoin and begin welded each other. Figure 6e shows the metal flow state at the inlet of the die orifice. The metal of welding chamber flows through the die bearing to form the final shape of the hollow profile. Once the metal extrudes through the die bearing, the flow velocity reaches maximum during the whole extrusion cycle. The small cavity region with relatively lower velocity compared to the other regions is the result of the additional friction resistance between the small mandrel and metal, which leads to a nonuniform velocity distribution on the cross-section of die exit and a warped profile, as shown above in Figure 5.

**Figure 7:**
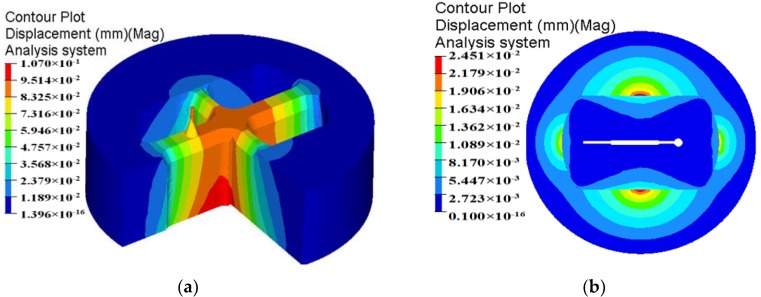
Displacement of the traditional designed die: (**a**) upper die and (**b**) lower die.

In billet-on-billet continuous extrusion, the porthole die usually works under high temperature, pressure and friction conditions, which easily causes porthole die elastic deformation or cracking. The deformation of mandrel and orifice seriously influences the accuracy and dimension of extruded profiles and the die life. By analyzing the die strength, the unreasonable design of porthole die could be found in advance and unnecessary waste of die is avoidable. In aspect of the aluminum profile with high length–width ratio and small cavity, for the upper die, strength checking mainly focuses on the small mandrel and port bridge, which are usually considered as simply supported girder beams. The mandrel with a diameter of 4.04 mm is especially liable to deflection and break during extrusion. Therefore, mandrel strength is one of the important factors to produce high accuracy profiles. For the lower die, there exists long cantilever beams in which deformation also occurs easily. The displacement distributions of the upper and lower dies for the initial design scheme are shown in Figure 7. It is clear that maximum displacement in the upper die is 0.107 mm, which lies at the intersection of port bridges. During extrusion process, the port bridges directly suffer higher compressive stress, which may lead to the port bridges collapse along the extrusion direction. The displacement in the end of the small mandrel is up to 0.0925 mm. It can be explained as follows: first, the small mandrel itself has bad rigidity and is easy to distortion. Second, there is lager pressure difference in radial and axial direction of small mandrel due to the nonuniform metal flow (See Figure 6). Deflection of the mandrel seriously affected the size accuracy of small cavity. Besides, the small mandrel is exposed to the outside of upper die and 26 mm higher than the die surface. During splitting or transporting, the small mandrel can easily deflect and break. As a result, we did not use it for the extrusion die. For the lower die, the maximum displacement is 0.0245 mm, which is located at the pier.

In order to improve the quality of profile and mandrel strength, the conventional die structure should be optimized. So it is crucial to find an appropriate way of porthole die optimization for balancing the metal flow and reducing mandrel deflection.

## 4. Redesign of Porthole Die

In the extrusion die design, there are many means to balance the metal flow in porthole die, such as by rationally designing the shape and position of portholes. Metal supplies can be balanced by adjusting the welding chamber height and welding angle; the metal flow resistance in porthole die can be controlled by adjusting the bearing length; the metal flow uniformity at the die exit can be improved greatly. The mandrel strength is mainly affected by the size of mandrel, the area of support surface, pressure in the welding chamber, pressure difference around mandrel, etc. The entrance angle of portholes, and the length and width of port bridges directly influence the strength of the bridges.

In this study, the modification schemes of porthole die were suggested as follows: Firstly, increasing the guiding angle in the entrance of portholes and adjusting the welding chamber from the lower die to the upper die. Then, the baffle plates are adopted in the welding chamber to encourage more metal to flow into the small cavity region. Finally, the bearing lengths are adjusted reasonably to obtain a more uniform exit velocity of profile.

### 4.1. First Step: Rearranging the Welding Chamber in Upper Die

**Figure 8:**
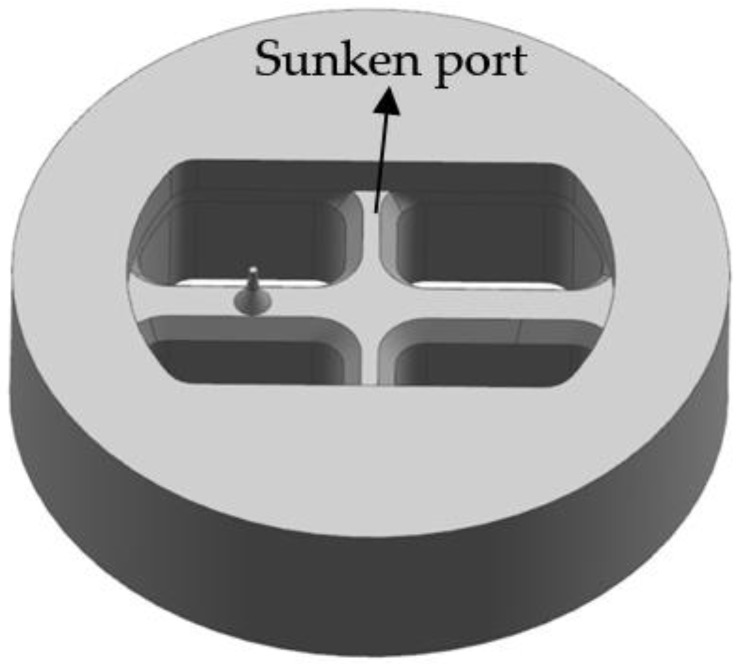
Geometric model of porthole die for the first modification scheme.

**Figure 9:**
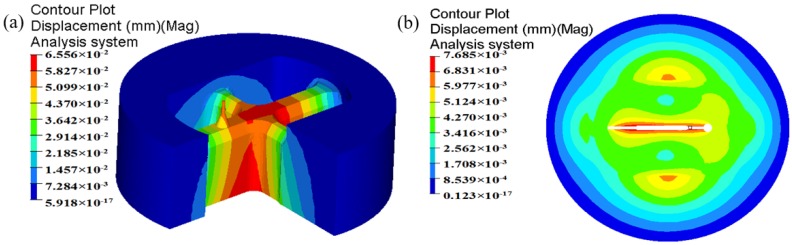
Displacement distribution of the first modification scheme: (**a**) the upper die and (**b**) the lower die.

**Figure 10:**
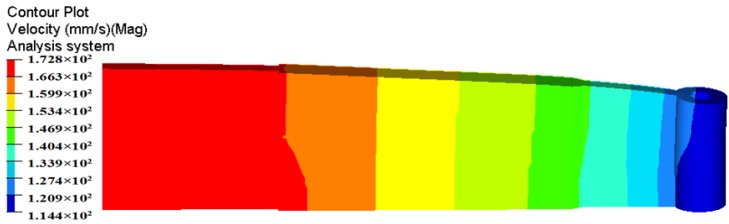
Velocity distribution in the extruded profile for the first modification scheme.

In the initial die design, the welding chamber was designed to in the lower die and the height of the welding chamber was 18 mm. With the rising of welding chamber height, the pressure around the mandrel increases, which leads to that the small mandrel is more easily deflected [7]. To improve the strength of upper die and prevent the mandrel from breaking in transporting or splitting, the welding chamber is redesigned to in the upper die. The port bridges were sunk with a value of 24 mm. There, the welding chamber height was decreased to 15 mm. On the other hand, the entrance angle of portholes was designed to 30° for guiding the material to easily flow into the portholes, resulting in decreasing the supporting force of port bridges and improving the die strength. Figure 8 shows the 3D geometric model of modified porthole die. Keeping the same process parameters to the initial extrusion process, the FE simulation was carried out with the modified porthole die. Figure 9 shows the displacement distributions of the upper and lower dies. In contrast to the simulation results of the initial porthole die, the die deflection is reduced significantly. The maximum displacement in the port bridges is decreased from 0.107 to 0.0655 mm, and the displacement of small mandrel is decreased from 0.0925 to 0.0518 mm. The maximum displacement of the lower die is located in the die orifice, the value is 0.00768 mm. Figure 10 shows the metal flow velocity distribution at the die exit obtained by the first modification scheme. In comparison with the initial die design, the maximum velocity is decreased from 182.3 to 172.8 mm/s, and the minimum velocity is increased from 111.0 to 114.4 mm/s. The SDV is decreased from 19.63 to 16.148 mm/s. However, there is still a large difference in metal flow velocity between the small cavity and other regions, which leads to the profile being bent and warped. Thus, it is necessary to take further measures to modify the die structure and balance the exit velocity of the profile.

### 4.2. Second Step: Introducing the Baffle Plates

**Figure 11:**
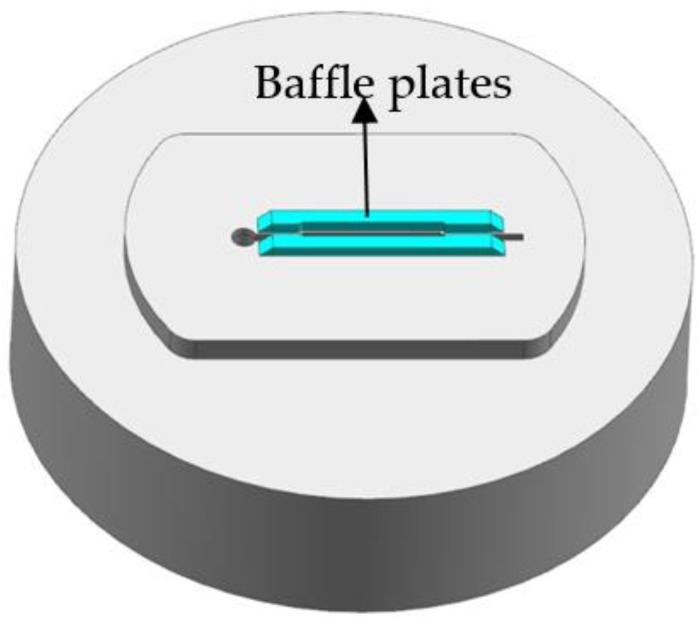
Location and shape of introducing the baffle plates.

**Figure 12:**
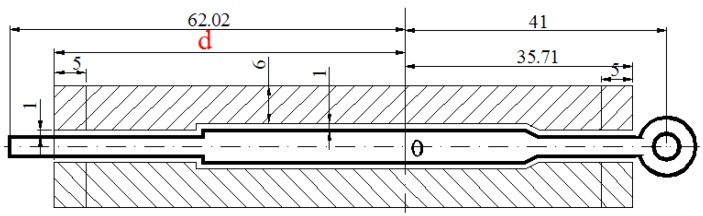
Detailed dimensions of baffle plates in the welding chamber (mm).

Based on the FE results of first modification scheme, the metal flow velocity in the part with small cavity is still relatively low, far less than the mean velocity at a value of 151.8 mm/s. Thus, additional baffle plates on the edge of the die orifice are introduced to increase the flow resistance in the regions in which metal flows faster than the small cavity region. Figure 11 shows the location and shape of introducing the baffle plates. Baffle plates play an important role for balancing the metal flow in the welding chamber. The main principle of baffle plates design is: where the metal flow out the die orifice is fast, a high baffle plate should be applied; in contrast, where the flow resistance is large, no baffle plate or a low one is used [13]. The location of baffle plates is an important factor that affects the velocity distribution of extruded profile at die exit, which is particularly discussed in this study. Figure 12 shows the detailed dimensions of baffle plates in the welding chamber. The height of baffle plates was 4 mm and the distance from the side edge of baffle plates to die orifice was 1 mm. In the part with small cavity, the length of baffle plates to the die center was 35.71 mm. In the solid end, the distance between the baffle plates and the die center is described as the variable *d*. The distance was adjusted from 55 to 65 mm, and the relevant FE analyses were carried out. The meshing principle, boundary conditions and bearings lengths in FE simulations kept consistency in each case to make effective comparisons.

**Figure 13:**
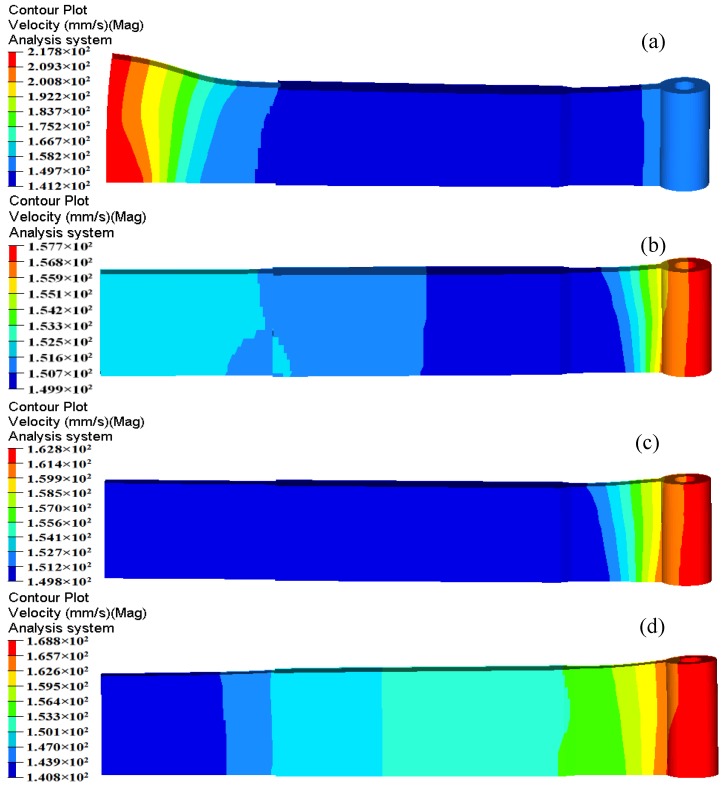
Velocity distribution in the extruded profiles at different positions of baffle plates: (**a**) *d* = 55 mm; (**b**) *d* = 57.5 mm; (**c**) *d* = 60 mm; (**d**) *d* = 65 mm.

The distortion and velocity distribution of extruded profiles under different distances *d* is shown in Figure 13. With the varying position of baffle plates, a considerable velocity difference is observed at die exit. When *d* is 55 mm, the metal flow velocity in the solid end is obviously faster than part with small cavity. A severe bending deformation occurs in the solid end. When *d* increases to 57.5 mm, a uniform exit velocity is obtained in the extrudate and little distortion is appeared. However, the small cavity has a relatively larger velocity comparing with the solid end in further increasing the value of *d*. Table 3 shows the comparison of maximum and minimum velocities, SDVs, and displacement of extrudate with different positions of baffle plates. It is clear that the maximum and minimum velocities for the Case 2 are 157.7 and 149.9 mm/s, respectively. The maximum velocity difference on the cross section of profile is 7.8 mm/s. The calculated SDV is 3.656 mm/s, indicating that the material flow in the welding chamber could be well regulated by effectively designing the locations and shapes of the baffle plates. For Case 2, the displacement of extrudate in the front end is 0.927 mm, which is much less than the other cases.

### 4.3. Third Step: Adjusting the Die Bearings

**Figure 14:**
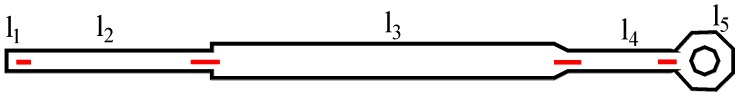
Uneven bearing lengths in the profile (mm).

**Figure 15:**
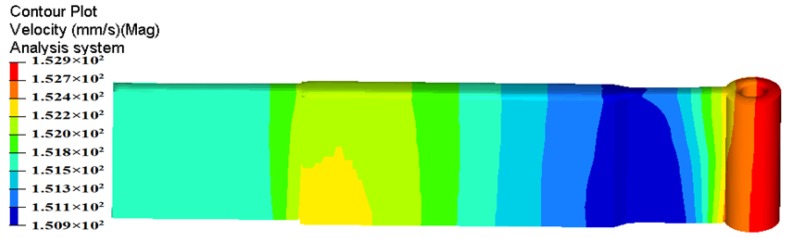
Velocity distribution of extruded profile with adjusting bearing lengths.

Die bearing plays a critical role in the production of high-performance extruded profiles. It is a common method in practice to balance the material flow and eliminate the extrusion defects by adjusting the lengths of die bearings. The design rule of die bearing is that a long bearing is used to increase additional frictional resistance in the zone with higher velocity, thereby reducing the velocity by which metal flows out the die orifice. On the contrary, a short bearing is utilized to decrease the fictional resistance. According to the information of exit velocity from Figure 13b, the bearing lengths of five regions in the profile were artificially adjusted reasonably, as is illustrated in Figure 14. Table 4 shows the bearing lengths for the initial and optimal dies. The effect of optimal design of die bearings on the velocity distribution of extruded profile at the die exit was examined. Figure 15 shows the deformation and velocity distribution of extruded profile with adjusting bearing length. By comparing Figure 13b and Figure 15, the velocity difference on the cross section of profile is further decreased to from 7.71 to 2 mm/s, and the SDV reduces from the initial 0.927 mm/s to 0.448 mm/s at present. Even more important, extrusion defects—such as distortion and warp on the extruded profile—are basically eliminated.

## 5. Comparison between the Initial and Optimal Porthole Dies

### 5.1. Metal Flow Pattern

**Figure 16:**
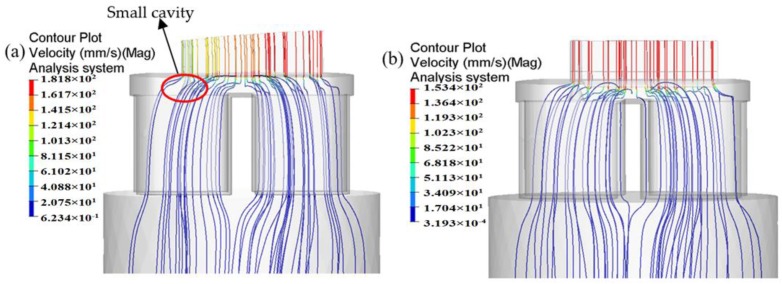
Comparison of metal flow patterns in porthole die: (**a**) the initial die and (**b**) the optimal die.

The metal flow pattern can be plotted by the streamlines panel of HyperView software using node or element vector fields. Figure 16 shows the metal flow patterns in the porthole die for the initial and optimal dies, which represent the flow paths of node particles from the billet to the final profile. It can be seen that, for the two die designs, there also exist multiple dead metal zones in the corner of the container and porthole die and between the sidewall and bottom of welding chamber. This may be explained by the strong sticking friction between the billet and extrusion tools under the non-lubricated conditions during porthole die extrusion. For the initial die design, the material flow through porthole die is severely not uniform. At the region with small cavity, the inlet edges of portholes and the bottom of port bridges, a few metal streamlines existed. At the die exit, the particles in the solid end have larger velocity, and they squeeze the particles in the part with small cavity where with low velocity. This squeezing action leads to a large distortion of extruded profile. Figure 16b shows the metal flow pattern for the optimal die design. By the three steps of die structure modifications, the particle lines at the die exit are almost parallel and all particles almost have the same velocity. This phenomenon indicates that the allocation of material and flow behavior in porthole die are almost reasonable.

### 5.2. Welding Pressure

**Figure 17:**
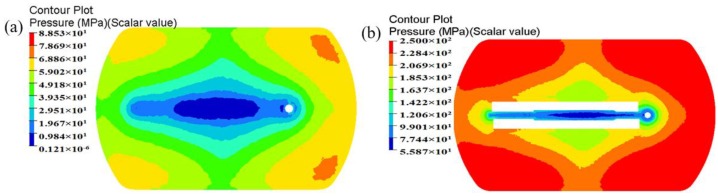
Comparison of the welding pressure distribution in middle height of the welding chamber (**a**) the initial die and (**b**) the optimal die.

In porthole die extrusion, the metal flowing through the portholes has to split up around the webs and then rejoin creating longitudinal welds that extend along the whole profile. The quality of weld seams is an important quality index for evaluating hollow aluminum profiles. Adhesive bonding under pressure plays a dominant role in the solid-state welding of the seams [6,25,26]. In recent years, the issue of longitudinal welding has attracted lots of interest from researchers, and several criteria were proposed for evaluating its quality, such as the maximum pressure criterion [27], pressure-time-flow criterion [28], and pressure-time-temperature-strain rate criterion [29]. It has been widely accepted that higher welding pressure is beneficial for improving welding quality. Akeret and Lee et al. [27,30] confirmed through experiments that the extruded profiles can be well welded when the welding pressure in the welding chamber exceeds the effective stress. Li et al. [31] concluded that when the peak welding pressure on the welding plane is three times beyond the yield stress of material, then can be recognized that the hollow profiles could weld. The peak welding pressure is almost located in the middle height of welding chamber [6,26]. The welding pressures in the middle height of welding chamber for the initial and optimal design schemes are shown in Figure 17. It can be seen that there exist four weld zones, i.e., four weld seams form in the surface of extruded profile. The pressure in the weld zones is obviously lower than that in the non-welded zones. For the initial design scheme, the mean pressure in four weld zones is 45.7 MPa, which is nearly equal to the effective stress of material. A lower welding pressure maybe lead to a undesirable welding strength. The mean welding pressure in four weld zones for the optimal die is 197.3 MPa, being 331.72% higher than that of the initial die. The ratio of mean welding pressure to the effective stress of the material is 4.5. Although the welding chamber height of the optimal die is decreased from 18 to 15 mm, the introduction of baffle plates increases greatly the flow resistance of material near the die orifice. Thus, the welding quality of extruded profile will be greatly improved.

### 5.3. Temperature Distribution

**Figure 18:**
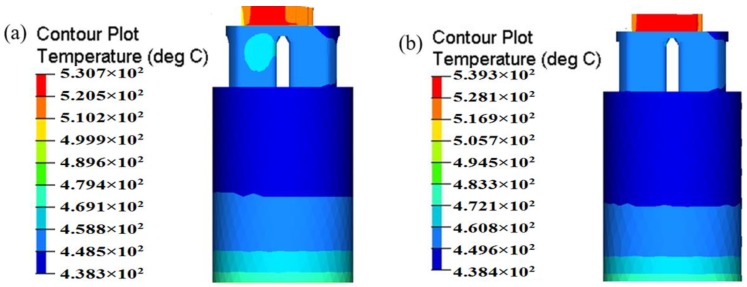
Comparison of the temperature distribution of billet (**a**) the initial die and (**b**) the optimal die.

The evolution of temperature in the billet during extrusion process is also an important indicator of attention to extrusion engineer. The temperature rise of extruded profile from the initial billet temperature may be beyond 100 °C [6]. Such strong thermal effects unavoidably affect the deformation behavior of material, extrusion load, and the microstructure of profile. In addition, the maximum temperature of extruded profile cannot exceed the critical temperature of AA6063-560 °C, since too high of a temperature may lead to hot tears outside the profile and low mechanical properties. Figure 18 shows the temperature evolution of billet during steady extrusion process for the initial and optimal design schemes. It is clear that the temperature of billet firstly drops gradually and then increases along extrusion direction. The temperature of billet in container decreases from the initial temperature of 480 °C to 438–480 °C due to the heat exchange with extrusion tools. With the proceeding of extrusion process, the billet temperature in the portholes increases to 458–469 °C as a result of heat generation in plastic deformation and friction. As the metal streams of portholes flow into the welding chamber and begins welded, more plastic deformation energy is converted into heat and increases the billet temperature. At the die exit, the billet temperature reaches its maximum, which is caused by large shear deformation in die orifice that generates considerable amounts of heat.

By analysis and comparison, a more uniform temperature distribution at the die exit is observed in the optimal die. This is beneficial to reduce the thermal deformation of profile and the difficulty of the subsequent heat treatment. This phenomenon may be attributed to the reasonable allocation of material and uniform material flow in porthole die for the optimal die design. As a result, the optimal die makes the distribution of the plastic deformation heat more even. The exit temperatures for the initial die and optimal die are both less than 560 °C, thus surface defects such as hot cracks or overburning can be effectively avoided in the extruded profile. The maximum temperature for the optimal die is 539.3 °C, being 8.6 °C higher than that of the initial die. The introduction of baffle plates increases the heat generation form plastic deformation and frictional contact, and results in a higher exit temperature.

### 5.4. Extrusion Load

The other key aspect is the influence of die structure modifications on the extrusion load, because the value determines whether the porthole die extrusion process can be carried out successfully. Besides, too high of an extrusion load will lead to an intolerable amount of wear for the porthole die, which is the major cause of die failure. Thus, the required extrusion load is an important parameter for evaluating the porthole die design. By a series of numerical simulations, the required extrusion loads for the different design schemes could be obtained as listed in Table 5. It can be seen that the required extrusion load for initial die design is relevantly low, the value is 10,617.3 KN. Through three kinds of die modifications, especially after introducing the baffle plates in the lower die, the steady extrusion load increases significantly due to the great increasing of the metal flow resistance in the welding chamber. However, the extrusion load for the optimal porthole die is 13,903.2 KN, which is still less than the maximum capacity of the 2000 ton extrusion press.

### 5.5. Die Displacement

**Figure 19:**
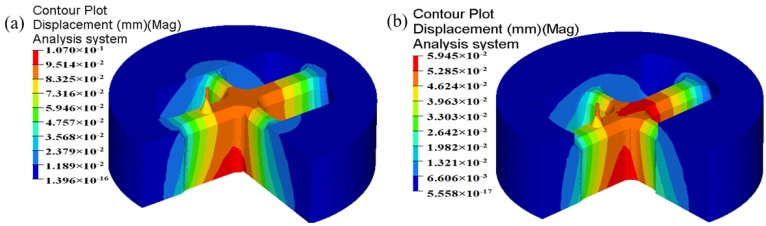
The displacement distribution in the upper die (**a**) the initial design scheme and (**b**) the optimal design scheme.

The displacement distribution of upper die for the initial and optimal design schemes is shown in Figure 19. It can be found that the maximum displacement in the upper die for the initial design scheme is 0.107 mm, which is located in the port bridges. After multi-step modifications, the maximum displacement in the upper die is reduced to 0.05945 mm. The displacement of mandrel is also decreased from 0.0925 to 0.04648 mm. In contrast to the initial design scheme, the elastic deformation of the optimal porthole die is quite small, thus the life of extrusion die is long. The dimensional tolerance caused by the elastic deformation of small mandrel does not affect the dimensional accuracy of extruded profiles. The optimal porthole die meets the production requirements of large aluminum profiles with high length–width ratio and small cavity.

## 6. Experimental Verification

**Figure 20:**
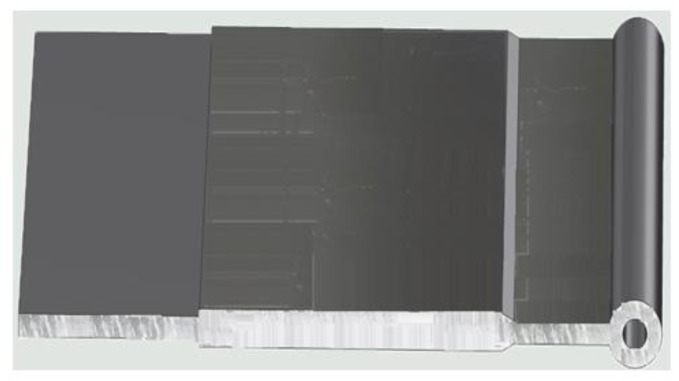
Qualified extrudate with electrophoretic coating through the optimal die.

**Figure 21:**
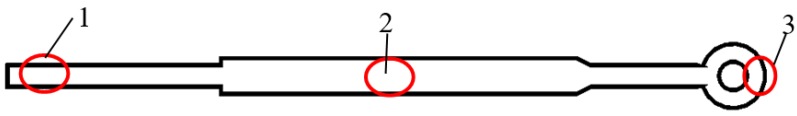
Distribution of the observation points on the cross section of the profile.

**Figure 22:**
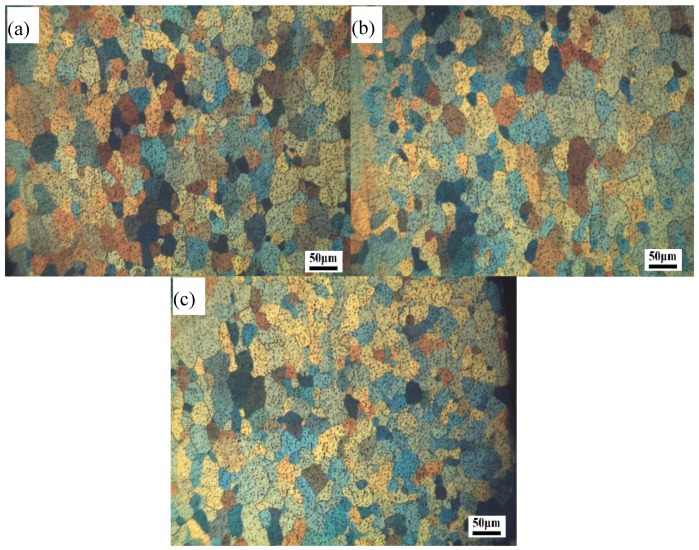
Optical micrographs in different observation points of (**a**) 1#, (**b**) 2#, and (**c**) 3# on the profile.

To validate the rationality of the optimization scheme, a real porthole die was manufactured and extrusion experiments were performed on a 2000 ton extruder. The process parameters—such as the temperature of initial billet and die, billet diameter, extrusion speed, etc.—adopted in the extrusion experiments were exactly consistent with those in simulations, as shown in Table 2. The extruded profile with electrophoretic coating is given in Figure 20. It can be seen that, by subsequent drawing, straightening, and correcting processes, no distinct extrusion defects such as bent or twist deformation along the length direction of profile is found. The measured and required thickness difference in the region of small cavity is only 0.1 mm, which meets the engineering requirements. Moreover, three typical positions on the cross section of profile were chosen to observe and analyze the microstructure (see Figure 21). For practical application, attention must be taken again to avoid overheating, as this is detrimental to the aluminum profile. The mechanical properties of extruded profiles are mainly determined by the grain structure. Figure 22 shows the optical micrographs of three different zones. It is clear that there are no remelting eutectics , local remelting that widens the grain boundaries, and remelting triangles in the intersection of three grains, which are the criteria to judge whether structure overheating occurs. It can be also seen that the deformation organization has basically disappeared and dynamic recrystallization occurs obviously during extrusion. The grain sizes in different positions of cross-section are basically equal. The main reason is that the optimized die allocates the material reasonably in porthole die and improves considerably the uniformity of metal flow velocity distribution at the exit of die, leading to a more uniform distribution of temperature on the cross section of the profile (see Figure 18b). The experimental results are basically consistent with the simulated ones, which indicates that the ALE formulation is effective and efficient in simulating the extrusion process with large deformation. This work could provide theoretical guidance in designing porthole dies for the kinds of large aluminum profile with high length–width ratio and small cavity.

## 7. Conclusions

In this study, the porthole die for producing an aluminum profile with small cavity was designed and optimized through numerical simulation. The steady state extrusion process was simulated using HyperXtrude software based ALE algorithm. The metal flow behavior in the porthole die for the initial die was revealed and the die strength was analyzed. Aiming at achieving a uniform flow velocity and enough die strength, three kinds of structure modifications for the porthole die were proposed. The metal flow pattern, welding pressure, temperature evolution, and die strength for the initial and optimal dies were analyzed and compared synthetically. The major findings of this study can be summarized as follows:

- The metal flow behavior in the porthole die at different stages of extrusion process for the initial die scheme is severely not uniform. The SDV at the die exit is 19.63 mm/s. The maximum displacement in the upper die and mandrel are 0.107 and 0.0925 mm, respectively.
- By three steps of die structure modifications, the SDV at the die exit is reduced to 0.448 mm/s. The distortion of the profile is avoided effectively. The mean welding pressure in the welding zones for the optimal die is 197.3 MPa, being 331.72% higher than that of the initial die. The maximum temperature of extrudate at the die exit for the optimal die is 539.3 °C, being 8.6 °C higher than that of the initial die. The maximum displacement in the upper die is decreased from 0.107 to 0.0656 mm, and the mandrel deflection is decreased from 0.0925 to 0.04648 mm.
- The good agreement between the simulation and experimental results shows the modification strategy of porthole die based ALE formulation is practicable and it can provide theoretical guidance for porthole die design of any other similar profiles.
- A design route of porthole die for aluminum profile with a small mandrel is proposed, including sunken port bridges to design the welding chamber in the upper die, increasing the inlet angle of portholes, adding the baffle plates, and adjusting the die bearings.

## Figures and Tables

**Table 1:** Physical properties of AA6063 aluminum alloy and AISI H13 steel.

Physical Properties	AA6063 Aluminum Alloy	AISI H13 Steel
Density (Kg/m^3^)	2700	7870
Young’s modulus (MPa)	68,900	210,000
Poisson’s ratio	0.3	0.33
Thermal conductivity (W/(m·K))	198	24.3
Specific heat (J/(kg·K))	900	460
Thermal expansion coefficient (1/K)	1.0 × 10^–5^	-

**Table 2:** Process parameters used in simulation.

Conditions	Values
Billet diameter (mm)	210
Billet length (mm)	350
Extrusion ratio	75.8
Extrusion speed (mm/s)	2
Billet temperature (°C)	480
Container and die temperature (°C)	430
Friction coefficient at billet/container and die	Sticking
Friction coefficient at billet/die bearing	0.3
Heat thermal coefficient between billet/container and die (W/(m^2^·°C))	3000

**Table 3:** Comparison of maximum and minimum velocities, SDVs, and displacements of extruded profiles at different position of baffle plates.

Design Schemes of Baffle Plates	Case 1	Case 2	Case 3	Case 4
Length of d (mm)	55	57.5	60	65
Max. velocity (mm/s)	217.8	157.7	162.8	168.8
Min. velocity (mm/s)	141.2	149.9	149.8	140.8
SDV	16.266	1.892	3.656	8.825
Displacement of profiles (mm)	6.657	0.927	1.499	3.318

**Table 4:** Bearing lengths for the initial and optimal dies.

Position	l_1_	l_2_	l_3_	l_4_	l_5_
Initial design (mm)	2	4	6.4	4.3	2.8
Optimal design (mm)	2.75	4.06	6.35	4.18	3.32

**Table 5:** Required extrusion load for the different design schemes.

Design Schemes	Initial Design	Modification Scheme 1	Modification Scheme 2	Modification Scheme 3
Extrusion load (KN)	10,617.3	10,140.6	13,718.4	13,903.2
